# KHNYN is a manganese-dependent endoribonuclease required for ZAP-mediated antiviral restriction

**DOI:** 10.1093/nar/gkaf1360

**Published:** 2025-12-17

**Authors:** Rebecca L Youle, Maria Jose Lista, Emma L Brudenell, Bradley Thompson, Clement Bouton, Elizabeth R Morris, Stuart J D Neil, Chad M Swanson, Ian A Taylor

**Affiliations:** The Francis Crick Institute, Macromolecular Structure Laboratory, 1 Midland Road, London NW1 1AT, UK; King’s College London, Department of Infectious Diseases, Floor 2 Borough Wing Guy’s Hospital, London SE1 9RT, UK; King’s College London, Department of Infectious Diseases, Floor 2 Borough Wing Guy’s Hospital, London SE1 9RT, UK; The Francis Crick Institute, Macromolecular Structure Laboratory, 1 Midland Road, London NW1 1AT, UK; AstraZeneca, The Discovery Centre, 1 Francis Crick Avenue, Cambridge CB2 0AA, UK; The Francis Crick Institute, Macromolecular Structure Laboratory, 1 Midland Road, London NW1 1AT, UK; King’s College London, Department of Infectious Diseases, Floor 2 Borough Wing Guy’s Hospital, London SE1 9RT, UK; The Francis Crick Institute, Macromolecular Structure Laboratory, 1 Midland Road, London NW1 1AT, UK; King’s College London, Department of Infectious Diseases, Floor 2 Borough Wing Guy’s Hospital, London SE1 9RT, UK; King’s College London, Department of Infectious Diseases, Floor 2 Borough Wing Guy’s Hospital, London SE1 9RT, UK; The Francis Crick Institute, Macromolecular Structure Laboratory, 1 Midland Road, London NW1 1AT, UK

## Abstract

Zinc finger antiviral protein (ZAP) is a cytoplasmic protein central to host innate immunity to viral infection. ZAP has no intrinsic catalytic activity but inhibits viral replication by binding to CpG dinucleotides in cytoplasmic viral RNA and recruiting other factors to inhibit protein synthesis and target the RNA for degradation. KHNYN is a ZAP-binding protein required for ZAP-restriction of CpG-rich viral genomes. It contains an extended diKH, PIN nuclease, and CUE^like^ domain, each of which are required for ZAP restriction of viral replication. Here, we report a structural, enzymological, and virological study of KHNYN’s essential PIN nuclease domain. Our crystal structure reveals an extended PIN domain (ex-PIN) containing a conserved N-terminal arm region required for domain stability and an active site tetra-Asp motif, which are both required for antiviral activity. Unlike the weak activity recently reported for the PIN domain, we demonstrate that the KHNYN ex-PIN domain is a highly active Mn^2+^-dependent single-stranded RNA endonuclease that cleaves with a preference for ApC, ApA, and UpA dinucleotides. These observations extend our view of KHNYN antiviral activity and suggest an unforeseen role for activation by manganese ions in the ZAP–KHNYN antiviral response.

## Graphical Abstract

**Graphical Abstract:**
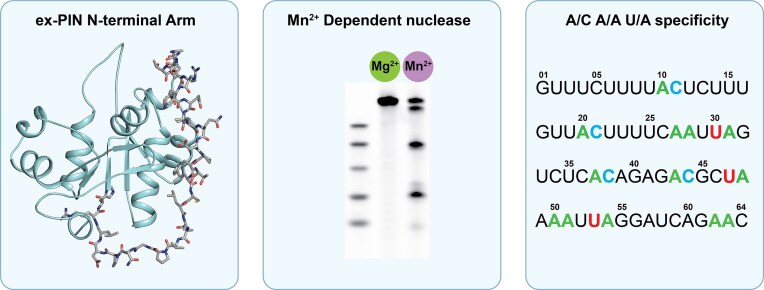

## Introduction

Zinc finger Antiviral Protein (ZAP, also known as ZC3HAV1 or PARP13) is an interferon-stimulated gene (ISG) found throughout the tetrapod lineage [1–4] that comprises a key component of the innate antiviral response [5]. ZAP restricts a wide variety of clinically important viruses including retroviruses, alphaviruses, Ebola virus, human cytomegalovirus, and SARS-CoV-2 [6]. In addition, ZAP also suppresses LINE-1 retrotransposon mobility through targeting of RNA intermediates [7, 8]. ZAP inhibits viral replication through binding viral RNAs containing clustered CpG dinucleotides to target them for degradation and thereby inhibiting their translation [4, 9–12]. However, as ZAP has no intrinsic enzymatic activity, it requires association with other cellular factors to form the ZAP antiviral complex [6, 13–18]. KHNYN was identified through yeast two-hybrid screening as a ZAP-interacting protein and is required for ZAP restriction of retroviruses [18] as well as other viruses [19, 20], but is not a core mammalian ISG [2]. It has two human paralogs, NYNRIN and N4BP1, that have diverse roles. NYNRIN evolved from a KHNYN gene duplication and may regulate placental development [21, 22]. N4BP1 is an ISG that is predominantly nucleolar [2, 23–26] and is best characterized for modulating the Nuclear Factor kappa-light-chain-enhancer of activated B cells (NF-KB) pathway [27–29]. N4BP1 has also been proposed to regulate HIV-1 gene expression and the E3 ubiquitin ligase Itch, and stability of specific cellular messenger RNAs (mRNAs) [30, 31]. N4BP1 shows some functional redundancy with KHNYN in inhibiting ZAP-sensitive viruses [19, 32, 33]. Both N4BP1 and KHNYN are required for TRIM25-mediated inhibition of lipid nanoparticle-delivered mRNA lacking mRNA-stabilizing and inflammation-reducing N^1^-methylpseudouridine modification [34].

Sequence analysis of human KHNYN identifies three structural domains: an N-terminal extended diKH (ex-diKH) domain, a central PIN domain and a C-terminal CUE^like^ domain [18, 35–37]. The ex-diKH domain has an unknown function and adopts an atypical orthogonal arrangement of the KH domains, found also in N4BP1, and unlike canonical diKH domains does not bind RNA [33, 38]. However, it is required for KHNYN–ZAP antiviral activity [18, 33]. The KHNYN CUE^like^ domain binds both ubiquitin and NEDD8 [37], contains a nuclear export signal [39] and more recently has been shown to also directly bind the ZAP RNA-binding domain [19, 38].

PIN domains form an extensive superfamily of nucleases, exhibiting diversity in reaction mechanisms, substrate specificity, biological functions, and taxonomic groups [40]. Phylogenetic analysis has demonstrated that the PIN domains of KHNYN and the closely related paralogs NYNRIN and N4BP1 [21] classify within the PRORP group [41] of the PIN superfamily [25, 40]. The catalytic cores of PIN domains invariably contain tetra-Asp motifs that structural studies have shown are required to co-ordinate metal ions [36, 42, 43]. Previous studies of the KHNYN PIN domain have shown that the tetra-Asp motif is required for KHNYN–ZAP antiviral activity [18, 19] and *in vitro* nuclease assays have reported a limited KHNYN nuclease activity with no detectable nucleobase specificity [35, 36, 38].

To characterize how the KHNYN PIN domain mediates viral RNA decay, we carried out biochemical studies, *in vitro* nuclease assays and determined the crystal structure of the complete KHNYN extended PIN domain. We identified a stabilizing N-terminal extended region, conserved in N4BP1, that is not present in the PIN domains of related ZC3H12 zinc-finger-containing proteins or in the previously reported KHNYN PIN domain structure [36]. By contrast to recent reports analysing KHNYN activity [35, 36, 38], our enzymatic data show that the KHNYN ex-PIN is a highly active and strictly manganese-dependent endoribonuclease. Additionally, our biochemical analysis reveals a specificity for cleavage at ApA, ApC, and UpA dinucleotides contrasting with the CpG binding specificity of ZAP. These observations provide an *in vitro* understanding of KHNYN endonuclease activity and suggest how ZAP–KHNYN activity might be regulated through both recruitment by ZAP to target RNAs and by sequestration of manganese ions to the active site to direct RNA hydrolysis.

## Materials and methods

### Cloning, protein expression, and purification

For expression in *Escherichia coli*, the DNA sequences coding for the human KHNYN PIN (residues P435–L591), KHNYN ex-PIN (residues V410–P594), and KHNYN ex-PIN-CUE^like^ (residues V410–F678) domains were amplified by polymerase chain reaction (PCR). KHNYN PIN was inserted into a modified pET22b(+) expression vector by restriction enzyme cloning (XmaI/XhoI) to produce an N-terminal MBP-tag and C-terminal His-tag fusion protein (MBP-PIN-His). KHNYN ex-PIN and ex-PIN-CUE^like^ were inserted into a modified pET24a(+) vector using ligation independent cloning to produce N-terminal StrepII-tagged fusion proteins. KHNYN ex-PIN D525A/D525A mutant was prepared from the parent construct using site-directed mutagenesis through PCR. All insert sequences were verified by DNA sequencing. The primer sequences for cloning and mutagenesis are presented in Supplementary Table S1.

MBP-PIN-His was expressed in the *E. coli* strain BL21(DE3). StrepII-ex-PIN, StrepII-ex-PIN(D524A/D525A), and StrepII-ex-PIN-CUE^like^ were expressed in the *E. coli* strain ArcticExpress (DE3). All cultures were grown at 37°C with shaking before protein expression was induced by addition of 0.4 mM Isopropyl β-D-1-thiogalactopyranoside (IPTG) to log phase cultures (*A*_600_ = 0.6). BL21(DE3) cells were incubated for a further 18 h at 18°C, and ArcticExpress (DE3) cells were incubated for a further 24 h at 13°C. Cells were harvested by centrifugation and resuspended in 5 ml of lysis buffer per g of cells before lysis with an Emulsiflex-C5 homogenizer, with three passes at 4°C. Details of all lysis and affinity purification buffers can be found in Supplementary Table S2.

Lysates were cleared by centrifugation for 1 h at 50 000 *× g* and 4°C. For MBP-PIN-His, lysate was applied to a 5 ml of HisTrap HP column (Cytiva) followed by extensive washing at 4°C before gradient elution of bound protein from final wash buffer to elution buffer over 10 column volumes. For StrepII-ex-PIN, StrepII-ex-PIN(D524A/D525A) and StrepII-ex-PIN-CUE^like^ lysates were applied to 10–20 ml of StrepTactin-XT affinity columns (IBA) followed by extensive washing at 4°C. Bound proteins were eluted from the column by step elution in final wash buffer supplemented with 50 mM D-biotin. For removal of N-terminal StrepII and MBP tags, GST-tagged HRV 3C-protease was added to affinity elution fractions and incubated overnight at 4°C. Proteins were then concentrated to ∼4 ml before injection onto a Superdex 75 (26/60) (GE) size-exclusion column equilibrated in GF buffer as a final purification stage. A 1 ml of GSTrap FF (GE) column was inserted in-line to remove GST-tagged HRV 3C-protease. Peak fractions, identified by Sodium dodecyl sulphate (SDS) gel electrophoresis, were concentrated and flash-frozen in liquid nitrogen in small aliquots at −80°C. Protein concentrations were determined from the UV/Vis absorbance spectrum using molar extinction coefficients at 280 nm (ε280) derived from the tryptophan and tyrosine content.

### NanoDSF

Protein stability was assessed through thermal shift assay monitored by nanoscale differential scanning fluorescence (NanoDSF) on a Prometheus NT.48 instrument (Nanotemper) employing standard capillaries. KHNYN ex-PIN domain samples (10 µl and 1.0 mg.ml^−1^) in buffer (50 mM Tris–HCl and 150 mM NaCl pH 7.5) with varying divalent metal ion composition (MnCl_2_, MgCl_2_, CaCl_2_, ZnCl_2_, CoCl_2_, and NiCl_2_ at 0.1, 1, 5, and 10 mM) were heated from 20 to 95°C at a constant rate of 1.5°C.min^−1^ whilst monitoring the fluorescence emission at 330 and 350 nm (λ_ex_ = 280 nm). PR.ThermControl v2.1.2 software was used to generate melting profiles from 350 nm/330 nm ratios against temperature and the first derivatives of these plots used to derive *T*_m_ apparent midpoints of melting transitions.

### RNA and DNA oligonucleotides

HPLC purified RNA and DNA oligonucleotides were synthesized by Horizon Discovery Biosciences Limited. RNA was deprotected according to manufacturer’s protocol, lyophilized and then resuspended in TE buffer (10 mM Tris pH 8.0 and 0.1 mM EDTA). DNA oligonucleotides were directly resuspended in TE. Oligonucleotide concentrations were determined from the *A*_260_ nm derived from the UV/Vis absorbance spectrum using extinction coefficients based on base sequence composition provided by the manufacturer.

### Nuclease assays

Nuclease assays were performed to evaluate the RNA degradation activity of ex-PIN. Certified nuclease free buffer components were from Invitrogen. RNaseZAP Wipes (Thermo Scientific) were used on all surfaces to prevent nuclease contamination. A variety of 5′ FAM-labeled ribo-oligonucleotides and deoxyribo-oligonucleotides (Supplementary Table S3) were employed in these assays. Typical time courses of RNA digestion were performed using 10 μM of RNA substrates in assay buffer [25 mM Tris–HCl pH 8.0, 150 mM NaCl, 10% (v/v) glycerol, 2.5 mM MnCl_2_, and 0.5 mM TCEP] and a reaction volume of 20 µl. In metal ion dependency and inhibition experiments, additional metals were added up to 10 mM or MnCl_2_ was replaced at 2.5 mM or by 10 mM EDTA. Heat inactivation of ex-PIN was performed by heating at 70°C for 5 min prior to its use in activity assays. RNase Inhibitor (Invitrogen Ambion™), when included, was at 40 units. Assays were performed over a time course of 0–180 min with ex-PIN or ex-PIN-CUE^like^ at 2–4 μM and ex-PIN (D524A/D525A) at 40 μM. For all assays, a no protein negative control of 10 μM RNA incubated at 37°C for the duration of the assay was included. Reactions were stopped by addition of 2× TBE–Urea sample buffer (Invitrogen) followed by incubation at 95°C. Products were resolved by denaturing gel electrophoresis on 15% TBE–Urea precast mini-gels (Novex) for 1 h at 180 V in 1× TBE buffer.

In DNA experiments, double-stranded DNAs were prepared at 50 μM in annealing buffer (10 mM Tris pH 8.0, 50 mM NaCl, 1 mM MgCl_2_, and 0.1 mM EDTA) by heating to 95°C for 5 min followed by slow cooling to room temperature. 50 nM of each DNA substrate was incubated in assay buffer with or without 500 nM ex-PIN for 1 h at 37°C in a reaction volume of 30 μl. Reactions were stopped by addition of 3 μl of stop buffer (166 mM EDTA, 3% SDS, and 6.7 mg.ml^−1^ Proteinase K) (Thermo Scientific) and heating to 50°C for 30 min followed by addition of 33 μl of 2× formamide sample buffer (95% formamide, 18 mM EDTA, and 0.025% SDS) and incubation at 95°C for 5 min. Products were separated by denaturing gel electrophoresis on a 20% TBE–Urea gels prepared with the SequaGel UreaGel System (National Diagnostics), according to the manufacturer’s protocol. The gel was pre-electrophoresed at 9 W in 1× TBE running buffer for 30 min prior to sample loading and further electrophoresis at 6 W for 40 min.

All gels were imaged with a Typhoon^™^ 9500 (Cytiva, USA) fluorescence molecular imager with the FAM-default sample detection method (λ_laser_ = 473 nm, λ_emission_ = 520 nm, filter LPB) at gains between 400 and 750 V. Integrated Band intensities were quantified using the ImageQuant TL Software (Cytiva).

### Crystallization and structure determination

Prior to crystallization, KHNYN ex-PIN was diluted to 3.1 mg.ml^−1^ with gel filtration buffer. Crystals were produced by sitting drop vapor diffusion at 18°C using a Mosquito^®^ crystal robot (SPT Labtech) to prepare 0.1 µl droplets containing a 2:1 ratio of protein solution to mother liquor. The best crystals were obtained from a mother liquor of 100 mM bis–tris–propane HCl pH 6.5, 200 mM NaF, and 20% (v/v) PEG 3350. For data collection, 30% (v/v) glycerol was added to the crystallization condition and crystals flash frozen in liquid nitrogen.

Diffraction datasets were collected on beamline i04 at Diamond Light Source, UK, using an X-ray wavelength of 0.9537 Å. Data were processed using the Xia2 DIALS pipeline [44]. Indexing and integration utilized XDS [45] or DIALS; point-group symmetry was determined with POINTLESS [46], isotropic scaling was carried out using AIMLESS [47], and structure factors generated using CTRUNCATE [48]. The ex-PIN structure was solved by molecular replacement using the program PHASER [49] implemented in the CCP4 interface [50] using a truncated Alphafold model [51] of KHNYN residues 435–594 as an initial search model. After molecular replacement and prior to any further refinement the initial model was removed and Buccaneer [52] employed to rebuild the entire map with input of the KHNYN ex-PIN sequence. Subsequently manual building within the program COOT 0.9.8.93 [53] was combined iteratively with refinement utilizing individual *B*-factors in Phenix 1.21.1 [54] to produce a final model for residues T411-R513, Y523-D543, E551-P594 (Chain A), and T411-K593 (chain B) refined to 2.35 Å with *R*_work_/*R*_free_ of 19.8/27.8. MolProbity [55] and PDB_REDO [56] were used to monitor and assess model geometry. The coordinates and structure factors have been deposited in the Protein Data Bank under accession number 9S2D.

### Structure and sequence-based analysis

Protein domain prediction was performed with AlphaFold3 [57] and Phyre2 [58]. PDB files from crystal structures were viewed and rendered in PyMOL 2.5.5 (Schrodinger, LLC). Electron density maps were visualized in COOT 0.9.8.93 [53]. Analysis of protein interfaces within crystal structures, was performed with PDB-PISA [59]. The DALI server [60] was used to perform PDB25/PDB90 and all against all structural alignment searches. Published and predicted protein structures were obtained from the RCSB PDB [61] and the AlphaFold DB [62, 63]. Primary amino acid sequences were aligned within LASERGENE DNASTAR using Clustal-W default settings and coloring [64].

### SEC-MALLS

Size-exclusion chromatography coupled to Multi-Angle Laser Light Scattering (SEC-MALLS) was used to analyze molar masses of ZAP RBD, KHNYN ex-PIN and KHNYN ex-PIN-CUE^like^ constructs and ZAP RBD binding to ex-PIN and ex-PIN-CUE^like^. Typically, 100 µl of samples of 0.5–2 mg ml^−1^ were applied to a Superdex 200 10/300 INCREASE GL column equilibrated in 20 mM Tris–HCl pH 7.8, 150 mM NaCl, 5 mM MgCl_2_, 0.5 mM TCEP, and 3 mM NaN_3_ at a flow rate of 1.0 ml.min^−1^. The scattered light intensity and protein concentration of the column eluate were recorded using a DAWN-HELEOS-II laser photometer and an OPTILAB-TrEX differential refractometer (dRI) (d*n/*d*c* = 0.186) respectively. The weight-averaged molecular mass of material contained in chromatographic peaks was determined using the combined data from both detectors in the ASTRA software version 7.3.2 (Wyatt Technology Corp., Santa Barbara, CA).

### Plasmids and cell lines for *in cellulo* analysis

HeLa and TZM-bl cells were maintained in high glucose DMEM supplemented with GlutaMAX (Thermo Fisher Scientific), 10% fetal bovine serum, 100 U/ml penicillin, and 100 µg.ml^−1^ streptomycin and incubated under 5% CO_2_ at 37°C. KHNYN CRISPR HeLa cells, HIV-1_NL4-3_ (pHIV-1_WT_), and HIV*_env(_*_86–561)_CpG (pHIV-1_CpG_) in pGL4 and the CRISPR-resistant pKHNYN-FLAG and pKHNYN (524A/525A) plasmids have been described previously [18, 65]. pKHNYN(∆410–434) was cloned by site-specific mutagenesis and pKHNYN(N4BP1PIN) was generated using HiFi assembly (New England Biolabs). The primers used (Supplementary Table S1) were purchased from Sigma–Aldrich. All PCR reactions were performed using Q5 High-Fidelity DNA polymerase (New England Biolabs).

### Transfections

HeLa CRISPR KHNYN cells were seeded in 24-well plates. Cells at 70% confluency were transfected using TransIT-LT1 (Mirus) at a ratio of 3 µl of TransIT-LT1 to 1 µg of DNA according to the manufacturer’s instructions. 0.5 µg of pHIV-WT or pHIV-CpG and the designated amount of CRISPR resistant pKHNYN-FLAG or mutants for a total of 1 µg of DNA were transfected. Twenty-four hours post-transfection, the culture media was replaced with fresh media. Forty-eight hours post-transfection, the HeLa cells were lysed in 2× Laemmli buffer. The culture supernatant was also collected, filtered through a 0.45 µm filter and virions were pelleted by centrifugation for 2 h at 4°C and at 20 000 × *g* through a 20% sucrose cushion in phosphate-buffered saline (PBS) and resuspended in 2× Laemmli buffer.

### TZM-bl infectivity assay

The TZM-bl indicator cell line was used to quantify the amount of infectious virus produced by the transfected HeLa cells [66–68]. Briefly, cells were seeded in 96-well plates and infected the following day with supernatants of transfected HeLa cells. Forty-eight hours post-infection, the TZM-bl cells were lysed, and β-galactosidase expression was measured using the Galacto-Star System following manufacturer’s instructions (Applied Biosystems) and quantified as relative light units per second using a PerkinElmer Luminometer.

### Analysis of protein expression by immunoblotting

Cell lysates and virion lysates were boiled at 95°C for 10 min and resolved on 8%–16% mPAGE Bis–Tris precast gels (Millipore). Proteins transferred onto nitrocellulose membranes (GE Healthcare) that were then blocked in 5% nonfat milk in PBS with 0.1% Tween 20. Primary antibodies were incubated overnight at 4°C followed by three washes in PBS with 0.1% Tween 20. Membranes were then incubated with the corresponding secondary antibody (1:5000 anti-rabbit HRP (Cell Signaling Technology, 7074), 1:5000 anti-mouse HRP (Cell Signaling Technology, 7076), 1:5000 anti-mouse IRDye 680RD (LI-COR, 926-68 070), and 1:5000 anti-rabbit IRDye 800CW (LI-COR, 926-32211) diluted in blocking buffer for 1 h at room temperature and then washed three times in PBS with 0.1% Tween 20. Proteins were visualized by LI-COR (Odyssey Fc) measuring secondary antibody fluorescence or using Amersham ECL Prime Western Blotting Detection reagent (GE Lifesciences) for HRP-linked antibodies with an ImageQuant (LAS8000 Mini). The following primary antibodies used at dilutions of: 1:50 HIV anti-p24^Gag^ [69] (mouse hybridoma), 1:3000 anti-HIV gp160/120 [Rabbit, ADP421; Centralized Facility for AIDS Reagents (CFAR)], 1:1000 anti-FLAG (DYKDDDDK, Rabbit, Cell Signaling, 14793), and 1:2000 anti-β-actin (Mouse, Abcam; Ab6276).

### Statistical analysis

Statistics from experimental replicates were determined using Prism10 (GraphPad Software inc.). Data are represented as mean with error bars equal to ± one standard deviation.

## Results

### The extended KHNYN PIN domain

**Figure 1.**
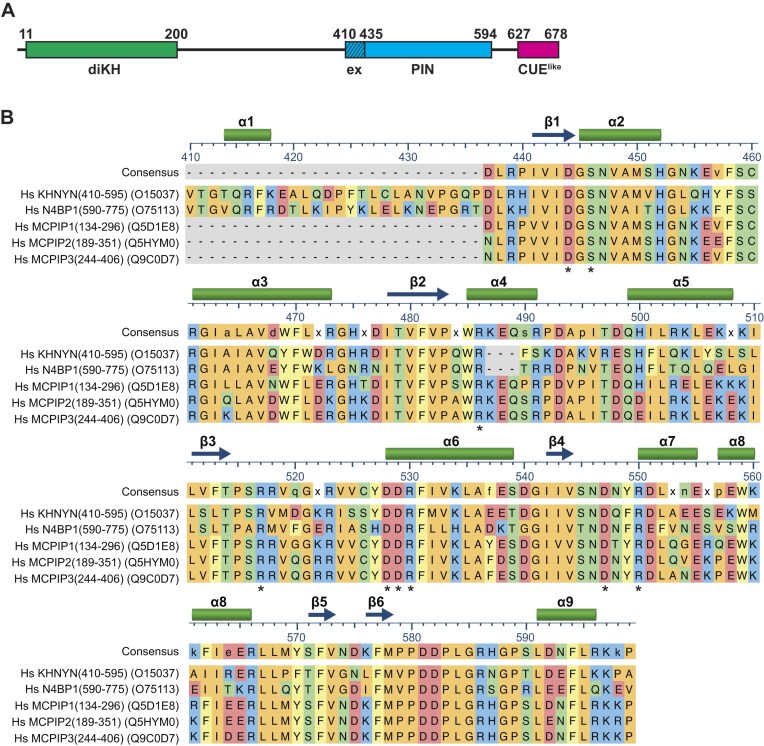
Structural Domains in ZAP and KHNYN. (**A**) Schematic representation of human KHNYN. Lines represent the primary sequence from N- to C-termini. The position of structural/functional domains are highlighted; green (di-KH domain: residues 11–200), cyan (ex-PIN domain: residues 410–594, extended region 410–435 is hashed), and magenta (CUE^like^ domain: residues 627–678). (**B**) Multiple sequence alignment using CLUSTAL-W of *Homo sapiens* KHNYN ex-PIN domain with PIN domains from N4BP1, MCPIP1 (ZC3H12A), MCPIP2 (ZC3H12B), and MCPIP3 (ZC3H12C). Uniprot accession numbers are shown on the right. The alignment is colored according to the CLUSTAL scheme [64], sequence numbers correspond to the KHNYN sequence. The positions of α-helix (cylinders) and β-strand (arrows) secondary structure elements in the KHNYN ex-PIN domain are shown above. The tetra-Asp motif and other residues conserved at the active site are indicated by the asterisks.

Our attempts to recover the core predicted KHNYN PIN domain (residues P435–P594) (Fig. 1A) by expression in *E. coli* resulted in only poorly soluble and largely aggregated protein samples. Therefore, using modelling in AlphaFold [57] and Phyre2 [58], combined with a sequence alignment of the PIN region with the close homologue N4BP1 and the PIN domain of ZC3H12 family members (Fig. 1B), we identified an N-terminal extension (residues V410–Q434) conserved in N4BP1 and KYNYN but absent from the ZC3H12 proteins. This extended PIN domain (ex-PIN) construct, KHNYN residues V410–P594, as well as an ex-PIN-CUE^like^ construct (residues V410–F678), had greatly improved solubility, were less aggregation prone and were readily purified from soluble extracts after *E. coli* expression. Both ex-PIN and ex-PIN-CUE^like^ constructs displayed a monomer molecular mass when analyzed by SEC-MALLS (Supplementary Fig. S1).

### Ex-PIN domain stability

**Figure 2.**
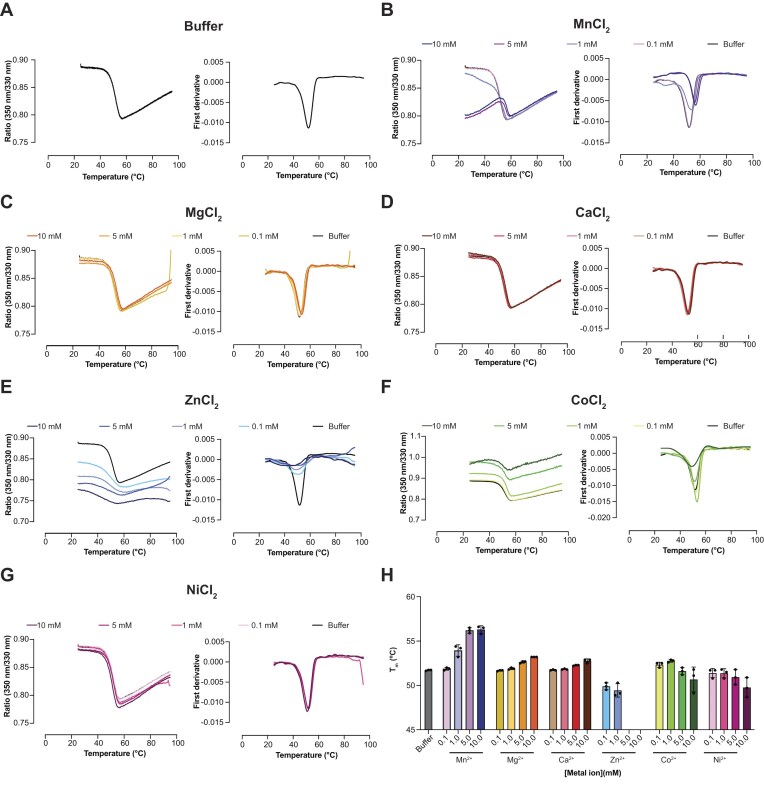
KHNYN stability. (**A–G**) Thermal denaturation of the KHNYN ex-PIN domain measured by DSF upon addition of different metal ions at 0.1, 1, 5, and 10 mM (A) buffer, (B) Mn^2+^, (C) Mg^2+^, (D) Ca^2+^, (E) Zn^2+^, (F) Co^2+^, and (G) Ni^2+^. Samples were heated at a rate of 1.5°. min^−1^ from 20 to 95°C whilst the fluorescence intensity was recorded at 330 and 350 nm (λ_ex_ = 280 nm). Curves from one experiment set are (left) the *F*_350_/*F*_330_ ratio derived from raw thermograms plotted against temperature at each divalent ion concentration and (right) the first differential plot of the ratio data. In each panel, the buffer only melting profile (black) is shown for comparison. (H) The average *T*_m_ plotted against metal ion concentration for three independent repeat experiment sets. Bars are color coded corresponding to metal ion identity in (A) to (G). Bar heights represent the mean value for *T*_m_ and error bars represent the standard deviation of the measurements from the three independent experiments.

To assess the folding and temperature stability of the ex-PIN domain, we measured its unfolding using nanoscale differential scanning fluorimetry (nanoDSF). Given the potential for the tetra-Asp motif to bind metal ions, we also tested the stabilizing/destabilizing effects of the expected active site metal ion, Mg^2+^, as well as the addition of variety of other divalent metal-ion chloride salts (Mn^2+^, Ca^2+^, Zn^2+^, Co^2+^, Ni^2+^). Metal ions were titrated from 0.1 to 10 mM to assess the impact on the thermostability of ex-PIN as determined from a melting transition midpoint (*T*_m_) shift. The unfolding curves, presented as first derivative plots, derived from the *F*_350_/*F*_330_ nm fluorescence emission ratio are shown in Fig. 2 and *T*_m_ values derived from these data are presented in Table 1. These data yield an apparent *T*_m_ for ex-PIN of 51.7°C derived for the apo protein measured in the absence of any divalent cation. The presence of increasing concentration of Co^2+^ and Ni^2+^ results in only slight destabilization of unfolding curves, with a reduction in *T*_m_ to 50.7°C and 49.8°C, respectively. Zn^2+^ causes a much more severe effect reducing the *T*_m_ around 3°C even at sub-millimolar concentration and results in protein precipitation at higher concentration, precluding reliable *T*_m_ measurement (Fig. 2E). By contrast, increasing concentration of Mg^2+^, Mn^2+^ and Ca^2+^ all resulted in *T*_m_ increases indicating all three of these divalent cations had a stabilizing effect on ex-PIN with Mn^2+^ providing the largest stabilizing effect raising the *T*_m_ to ~56.0°C.

**Table 1.** *T*_m_ of ex-PIN with addition of divalent metal ions

Metal ion	*T*_m_^a^	*T*_m_ (°C), 0.1 mM	*T*_m_ (°C), 1 mM	*T*_m_ (°C), 5 mM	*T*_m_ (°C), 10 mM	Δ*T*_m_^‡^ (°C), 10 mM^‡^
Buffer only	51.7 ± 0.1^†^	–	–	–	–	
Mn^2+^	–	51.9 ± 0.2	53.9 ± 0.6	56.2 ± 0.3	56.3 ± 0.4	+4.6 ± 0.4
Mg^2+^	–	51.7 ± 0.1	51.9 ± 0.1	52.6 ± 0.1	53.2 ± 0.03	+1.5 ± 0.1
Ca^2+^	–	51.7 ± 0.1	51.8 ± 0.1	52.2 ± 0.05	52.8 ± 0.2	+1.1 ± 0.2
Zn^2+^	–	49.4 ± 0.4	49.4 ± 0.8	n.d.	n.d.	n.d.
Co^2+^	–	52.3 ± 0.3	52.8 ± 0.2	51.6 ± 0.4	50.7 ± 1.4	−1.0 ± 1.4
Ni^2+^	–	51.4 ± 0.5	51.4 ± 0.5	50.9 ± 0.9	49.8 ± 1.1	−1.9 ± 1.1

^a^ *T*_m_ measured in buffer without addition of divalent metal ions; ^†^mean value and standard deviation from at least three independent measurements; ^‡^*T*_m_ difference between measurements at 0- and 10-mM divalent metal ion; n.d. protein precipitation precluded measurement.

### Human KHNYN ex-PIN is a Mn^2+^-dependent ssRNA endoribonuclease

**Figure 3.**
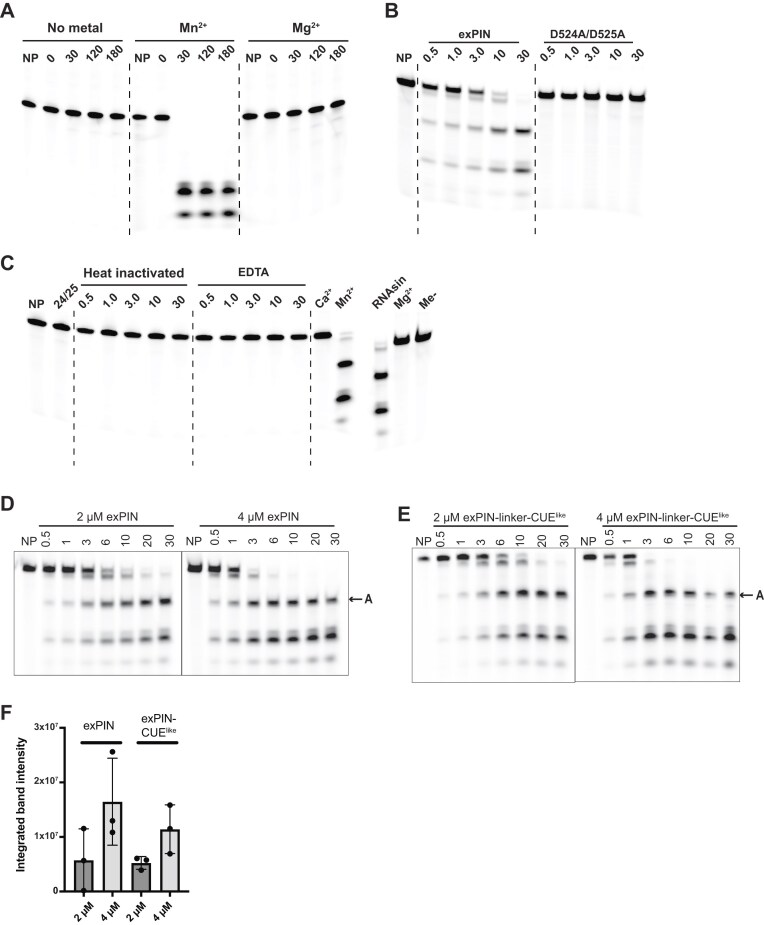
KHNYN endonuclease activity. (**A**) Divalent metal ion dependency of ex-PIN RNA hydrolysis. 10 µM 33mer 5′-FAM-labeled U-rich RNA incubated with 4 µM ex-PIN and 2.5 mM of the indicated divalent metal ions for 0 to 180 min. Substrate and hydrolysis products were separated by urea denaturing gel electrophoresis and visualized by fluorescence imaging. Reaction incubation times are indicated, NP, no protein control. (**B**) ex-PIN catalytic residues. 10 µM 33mer 5′-FAM-labeled U-rich RNA was incubated with 4 µM ex-PIN or 40 µM ex-PIN (D524A/D525A) for 0 to 30 min. Products were separated and visualized as in (A). (**C**) Abolition of ex-PIN endonuclease activity. 10 µM 33mer 5′-FAM-labeled U-rich RNA was incubated for 0.5 to 30 min with 4 µM ex-PIN either prior heat-treated at 70°C for 10 min or with 10 mM EDTA. NP; no protein control; 24/25, negative control 30-min incubation with 40 µM ex-PIN (D524A/D525A). Ca^2+^, Mn^2+^, RNase-inhibitor, Mg^2+^, Me-; 30-min reaction containing 2.5 mM CaCl_2_, MnCl_2_, MnCl_2 _+ RNase inhibitor (40 units), MgCl_2_, or no metal respectively. Products were separated and visualized as in (A). (**D** and **E**) ex-PIN and ex-PIN-CUE^like^ concentration dependency of hydrolysis reaction. Hydrolysis time courses (0.5–30 min) of 10 µM 33mer 5′-FAM-labeled U-rich RNA incubated with 2.0 and 4.0 µM (D) ex-PIN or (E) ex-PIN-CUE^like^. Substrate and products were separated and visualized as in (A). Tracks are labeled with times of incubation (min); NP, no protein control, product-A, 20 mer product resulting from an early cleavage. (**F**) quantitation of product-A at 1-min incubation. The integrated band intensity is plotted at each ex-PIN and ex-PIN-CUE^like^ concentration. Bars are average integrated intensity and error bars represent the standard deviation of data from three independent experiments. The amount of early product-A doubles with a 2-fold increase of enzyme indicating linear dependences of the reaction over this concentration range.

Previous studies demonstrating KHNYN PIN domain enzymatic activity using Mg^2+^ as the divalent cation and employing high enzyme to substrate ratio single turnover conditions [35, 36, 38] demonstrated largely incomplete RNA digestion suggesting that KHNYN exhibited only low enzyme activity. However, given the modest but reproducible effect we observed on protein stability upon addition of either Mg^2+^ and Mn^2+^, we investigated the metal ion dependency for KHNYN ex-PIN activity using steady-state nuclease assays employing a 5′-FAM-labeled 33mer U-rich substrate RNA substrate (Supplementary Table S3). These data (Fig. 3A) show that over a time course of ex-PIN incubation up to 180 min in buffer containing no metal or 2.5 mM MgCl_2_, the RNA migrates at the same molecular weight as the untreated RNA control and there is no evidence of degradation. This indicates that under these conditions, the protein lacks nuclease activity. By contrast, when 2.5 mM MnCl_2_ is included, there is near complete hydrolysis of the RNA, with two product species bands migrating at lower molecular weights compared to the untreated control and 0-min time point. The hydrolysis appears to be complete by 30 min as no further digestion of these products occurs, suggesting that these short products are now refractory to ex-PIN hydrolysis activity, either as they are too short, or the sequence composition is incompatible with ex-PIN nuclease activity. By comparison with previous single turnover assays employing high enzyme to substrate ratio [38], the cleavage we observe under steady-state conditions has substantially higher specific activity. Moreover, given the strong pause points and that there is no evidence of a ladder of products, this also supports the idea that ex-PIN possesses endonuclease rather than exonuclease activity.

The tetra-Asp motif of the ex-PIN KHNYN comprises residues D443, D524, D525, and D543. Mutations at D443 and 524/525 have been shown to severely diminish or abolish KHNYN antiviral activity, respectively [18, 19]. Therefore, to test that the observed nuclease activity was not due to a contaminating *E. coli* or exogenously introduced activity, D524A and D525A substitution mutations were introduced into the ex-PIN domain. Comparative nuclease time course assays with wild-type KHNYN ex-PIN and the D524A/D525A mutant are shown in Fig. 3B. These data show that over the reaction course with ex-PIN in buffer containing 2.5 mM MnCl_2_, RNA hydrolysis is apparent with faster migrating bands appearing compared to the untreated control. Hydrolysis is also time dependent, with faster migrating species increasing in intensity with time. In contrast, no observable hydrolysis occurs in assays employing the ex-PIN (D524A/D525A) mutant. The substrate RNA remains intact with no evidence of degradation, supporting the notion that when the catalytic residues of ex-PIN are mutated, the protein lacks nuclease activity under conditions which would normally lead to RNA hydrolysis.

To further characterize ex-PIN nuclease activity, we tested the impact of heat treatment at 70°C for 10 min or the addition of 10 mM EDTA on catalytic activity. Additionally, as nanoDSF demonstrated that CaCl_2_ also had a mild stabilizing effect on ex-PIN, 2.5 mM CaCl_2_ was substituted for MnCl_2_ to assess if this divalent metal ion could also activate ex-PIN nuclease activity. In the heat-treated ex-PIN samples, the substrate RNA migrates at the same molecular weight as the untreated RNA and the RNA in the negative control ex-PIN (D524A/D525A) sample (Fig. 3C), demonstrating that heat denaturation effectively inactivates ex-PIN nuclease activity. Similarly, in assays containing 10 mM EDTA, the RNA shows no evidence of degradation. This demonstrates that sequestration of MnCl_2_ divalent metal cations by the EDTA abolishes nuclease activity, further supporting that ex-PIN nuclease activity is MnCl_2_ dependent. Replacement of MnCl_2_ with CaCl_2_ also results in the loss of activity suggesting that like Mg^2+^ and Ca^2+^ cations are also unable to support substantial ex-PIN nuclease activity. A further sample, containing the Ambion^™^ RNAse A inhibitor shows a similar level of hydrolysis as with MnCl_2_. This showed that knockout of background nucleases by the inhibitor does not lead to any loss of nuclease activity and is further evidence that activity seen is solely due to ex-PIN. Analysis of the dependence of the RNA hydrolysis rate on ex-PIN concentration (Fig. 3D) and that of the longer ex-PIN-CUE^like^ construct (Fig. 3E) demonstrated no differences in the hydrolysis banding pattern, a comparable rate and a linear proportionality with enzyme concentration (Fig. 3F). These data further support the notion that ex-PIN alone supports Mn^2+^ stimulated RNA hydrolysis and that there is no influence on activity or sequence preference from the CUE^like^ domain.

### KHNYN ex-PIN substrate and sequence specificity

**Figure 4.**
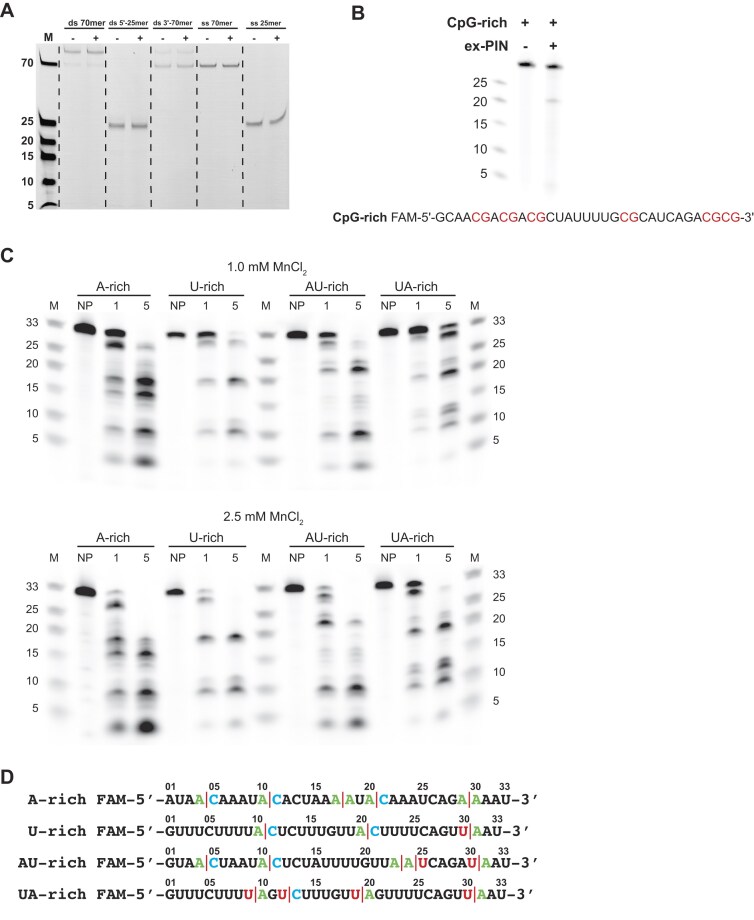
KHNYN endonuclease specificity. (**A**) ex-PIN nucleic acid specificity. 50 nM 5′-FAM-labeled DNA substrates; blunt double-stranded 70mer (ds 70mer), double-stranded 25mer – 5′ overhang (ds 5′-25mer), double-stranded 70mer – 3′ overhang (ds 3′-70mer), single-stranded 70mer (ss 70mer), and single-stranded 25mer (ss 25mer) incubated with (+) and without (−) 500 nM ex-PIN at 37°C for 60 min. Products were separated by urea denaturing gel electrophoresis and visualized by fluorescence imaging. M, ss DNA size markers. Slower migrating bands in double-stranded samples lanes are residual nondenatured duplex DNA. (**B**) ex-PIN substrate RNA sequence specificity, CpG dependence. (Upper) 10 µM 33mer 5′-FAM-labeled CpG-rich RNA incubated with or without 4.0 µM ex-PIN for 10 min at 37°C. RNAs were separated by urea denaturing gel electrophoresis alongside FAM-labeled RNA size markers, lengths indicated, and visualized by fluorescence imaging. (Lower) Sequence of CpG rich RNA derived from the recoded region in HIV-CpG, the CpG di-nucleotides are highlighted in red. (**C**) ex-PIN substrate RNA sequence specificity. 10 µM 33mer 5′-FAM-labeled A-rich, U-rich AU-rich and UA-rich RNAs were incubated with 4.0 µM ex-PIN and 1.0 mM (upper) or 2.5 mM (lower) MnCl_2_. M, RNA size markers; NP, no protein control; 1 and 5, incubation time (min). Substrate and products were visualized as in (B). (**D**) Endoribonucleolytic cleavage mapping. Cleavages within sequences derived by comparison with size markers are mapped onto the A-rich, U-rich AU-rich, and UA-rich RNAs. Cleavage points are represented by red lines with the surrounding dinucleotide highlighted in color. Strong cleavage points are observed between AC, AA, and UA dinucleotides.

To assess the range substrates that could be hydrolysed by the ex-PIN we conducted Mn^2+^-stimulated nuclease assays with a selection of single-stranded DNAs and double-stranded DNAs comprising blunt-ended as well as 5′- and 3′-overhang duplexes (Supplementary Table S3). These experiments, carried out under conditions of excess enzyme to identify even very weak activity, showed that in each of the assays (Fig. 4A), the DNA migrates at the same molecular weight as the untreated controls revealing that ex-PIN lacks nuclease activity against this selection of DNA substrates under conditions that readily hydrolyze an RNA substrate.

To examine ex-PIN sequence specificity, we also carried out nuclease assays with RNA substrates comprising CpG-rich, A-rich, U-rich, AU-rich, and UA-rich sequences (Supplementary Table S3). These data show that whilst the CpG-rich oligo was largely resistant to digestion (Fig. 4B), the A-rich, U-rich, AU-rich, and UA-rich sequences were all cleaved endonucleolytically to yield discrete bands (Fig. 4C). The cleavage sites of the ex-PIN can then be mapped using size standards to reveal potential cleavage-site specificity. Analysis of these cleavage products (Fig. 4D) revealed out of 17 mapped cleavages, 15 occurred between ApC, ApA, and UpA dinucleotide pairings, 7/17, 3/17, and 5/17, respectively. These data, whilst not exhaustive, suggest ex-PIN nuclease activity exhibits a preference for cleavage at ApC, ApA, and UpA dinucleotides whilst selecting against CpG sites in the CpG-rich sequence and cleavage within poly-U stretches present in the RNAs.

### Metal ion inhibition of KHNYN ex-PIN nuclease activity

**Figure 5.**
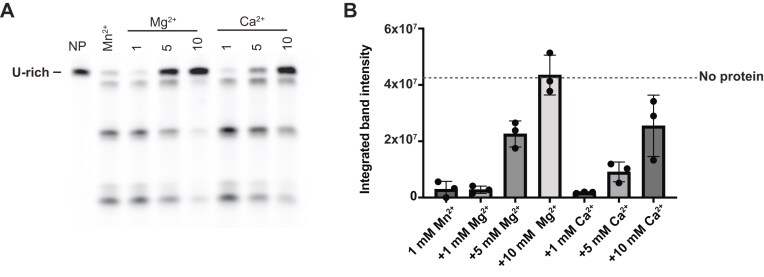
KHNYN endonuclease metal-ion inhibition. (**A**) ex-PIN inhibition by divalent metal ions. 10 µM 33mer 5′-FAM-labeled U-rich RNA incubated with 4 µM ex-PIN and 1.0 mM MnCl_2_ with addition 1.0, 5.0, and 10 mM MgCl_2_ or CaCl_2_. Samples were taken from reaction time courses at 5.0 min, substrate and products separated by urea denaturing gel electrophoresis and visualized by fluorescence imaging. NP, no protein control, Mn^2+^, 5-min reaction with no additional metal ions. (**B**) Quantitation of substrate (U-rich RNA) intensity derived from (A). The integrated band intensity determined for remaining substrate in the Mn^2+^ and reactions containing additional Mg^2+^ and Ca^2+^ ions after 5-min incubation is plotted against metal ion concentration. Bars are average integrated intensity and error bars represent the s.d. of data from three independent experiments. Dashed line represents the average value of U-rich RNA in the no protein control.

Given the lack of digestion observed when either Mg^2+^ and Ca^2+^ ions were included in in our hydrolysis reactions, we postulated that under these conditions these divalent cations may potentially be inhibitory to ex-PIN endonuclease activity. To test this notion, a Mn^2+^ stimulated ex-PIN nuclease assay, using the 5′-FAM-labeled U-rich RNA containing three primary cleavage sites (Fig. 4D), was conducted in the presence of increasing concentration of Mg^2+^ or Ca^2+^ ions. Samples of the reaction products taken after five minutes incubation were resolved by urea denaturing gel electrophoresis and visualized to determine the extent of hydrolysis (Fig. 5A). These data showed that whilst addition of equimolar (1 mM) Mg^2+^ or Ca^2+^ to Mn^2+^ stimulated reactions had little or minimal effects on the RNA hydrolysis, increasing Mg^2+^ or Ca^2+^ concentration to 5–10 mM reduced the amount of digestion products substantially. Quantitation of these reactions by integration of the substrate band intensities observed at different metal ion concentrations (Fig. 5B) showed that inclusion of Mg^2+^ at 5 mM reduced the amount of substrate hydrolysis around 10-fold and at 10 mM resulted in near complete inhibition with the substrate remaining at the same level as in the no enzyme control. Similarly, Ca^2+^ also reduced ex-PIN digestion of RNA substrate with ~50% of the substate still present when 10 mM Ca^2+^ was included in reactions. These data show that not only are Mg^2+^ and Ca^2+^ unable to support ex-PIN endonuclease activity, in fact, they are inhibitors of ex-PIN activity potentially acting competitively with Mn^2+^ at the active site to prevent formation of catalytically competent enzyme–metal ion complex.

### Crystal structure of the human KHNYN ex-PIN domain

**Figure 6.**
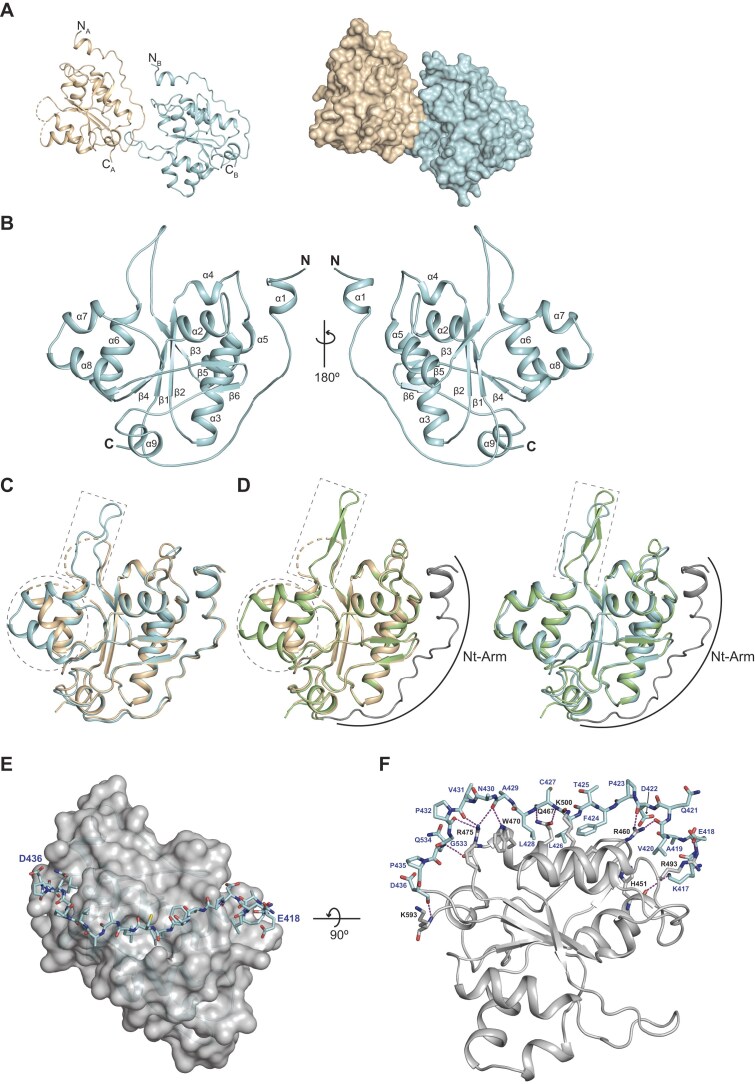
Structure of the KHNYN ex-PIN domain. (**A**) The dimer asymmetric unit of the KHNYN ex-PIN domain crystal structure. The protein backbone of the two ex-PIN protomers, Monomer-A (wheat) and Monomer-B (cyan) are shown in cartoon representation (left) and in surface (right). N- and C-termini of each monomer are labeled in the left-hand panel. (**B**) Secondary structure of ex-PIN. The protein backbone of the ex-PIN Monomer-B is shown in cartoon, two views rotated by 180°. Secondary structure α-helices and β-strands are labeled sequentially from the N- to C-terminus. (**C**) 3D DALI structural alignment of ex-PIN Monomer-A and Monomer-B (RMSD 1.4 Å; 167 of 183 Cα atoms). The β3–α6 loop (residues 510–524) and α7–α8 (residues 540–562) of the α6–α8 helical wing that have alternative conformations are boxed in gray. (**D**) 3D DALI structural alignment of the PIN structure from (PDB: 8WZC) with ex-PIN Monomer-A (RMSD 1.7 Å; 146 of 183 Cα atoms) (left) and Monomer-B (RMSD 1.2 Å; 161 of 183 Cα atoms) (right). Structures are shown in cartoon representation, PIN (green), ex-PIN Monomer-A (wheat), and Monomer-B (cyan). The ex-PIN Nt-Arm (residues 410–436), absent in the PIN structure, is highlighted and colored gray in each superposition. Gray boxed areas highlight regions of structural divergence. (**E**) The Nt-Arm–PIN domain interface. The PIN domain is shown in gray semi-transparent surface with the Nt-Arm residues, E418 to D436, that traverse the PIN domain shown in stick representation. (**F**) Close-up of the Nt-Arm–PIN molecular interface. Ex-PIN is viewed at 90° to (E). The backbone of the PIN domain is shown in gray cartoon. The Nt-Arm shown in sticks colored by atom type with residues labeled. Nt-Arm–PIN hydrogen bonds are displayed as dashed lines (purple) with PIN residues making interactions also shown in stick representation colored by atom type.

A previous study reported the crystal structure of the KHNYN PIN domain (residues D436–A595) [36] that lacked the evolutionarily conserved N-terminal extension. Therefore, to understand the contribution of this N-terminal extension to protein folding, stability, and catalysis, we determined the ex-PIN crystal structure that includes the N-terminal extension sequence. The data collection and structure refinement statistics are presented in Table 2. Within the structure, residues encompassing T411–P594 were well ordered, which includes residues T411–D436 of the N-terminal extension. The asymmetric unit comprised two copies of the ex-PIN (Fig. 6A) that pack to bury a surface area of 446 Å^2^, although there is no indication of dimerization for the ex-PIN at up to 2 mg ml^−1^ concentration in solution as determined by SEC-MALLS (Supplementary Fig. S1). The domain comprises a mixed α/β fold containing nine α-helices and six β-strands (Fig. 6B). The ex-PIN N-terminal sequence forms an N-terminal arm (Nt-Arm) comprising a short helix, α1 and an extended strand that packs against the core PIN domain. Further secondary structures are arranged in two helical subdomain wings (α2–α5 and α6–α8), a central β-sheet (β1–β4), small orthogonal sheet (β5–β6), and a C-terminal short helix, α9. The copies structurally superpose well with a RMSD of 1.4 Å over 167 of 183 Cα positions but with two local conformational differences (Fig. 6C). The first in the large loop that intersperses β3 and α6 that is disordered in Monomer-A. The second in the (α6–α8) helical wing, where in Monomer-A α7 is disordered and α8 is rotated by 25° relative to its position in Monomer-B.

**Table 2.** X-ray data collection and structure refinement statistics

	KHNYN (V410–P594)
Data collection	Diamond I04
Space group	P4_1_
Cell dimensions*a, b, c* (Å)*α, β, γ* (°)	54.72.54.72 110.8590, 90, 90
Wavelength (Å)	0.9537
Resolution (Å)	54.72–2.35 (2.39–2.35)^a^
Unique reflections	13 672 (664)
*R*_meas_ (%)	22.0 (61.8)
*R*_pim_ (%)	6.2 (17.8)
*CC_1/2_*	0.995 (0.875)
*I/σ(I)*	5.5 (0.7)
Completeness (%)	100 (97.6)
Multiplicity	12.6 (11.9)
	
Refinement	Phenix 1.21.1
Resolution (Å)	54.72–2.35 (2.58–2.35)
Refl working/free	13 636/628 (3231/165)
*R*_work_ */R*_free_/Test set size (%)	19.8/27.8/4.6 (28.1/34.3/4.9)
No residues/atoms	
Protein	345/2741
Water	45
All	2786
*B-*factors (Å^2^)	
Wilson	43.6
Protein	50.9
Water	49.0
Geometry	
RMSD Bond lengths (Å)	0.009
RMSD Bond angles (°)	1.139
Ramachandran Outliers (%)	0.0
Ramachandran Allowed (%)	4.8
Ramachandran Favored (%)	95.2
Molprobity score (*N* number/percentile)	2.11 (9377/87th)
PDB code	9S2D

^a^Values in parentheses refer to the highest resolution shell

Structural alignment using the DALI server [60] of the two ex-PIN copies with the published Mg-RNA bound KYNYN PIN domain [36] not containing the Nt-Arm (PDB: 8WZC) reveal a high degree of similarity within the core PIN domains (RMSD 1.2–1.7 Å). However, in addition to the absence of the Nt-Arm in the PIN structure, the β3–α6 loop conformation differs in all three structures. Moreover, the conformation of the α6–α9 helical wing in the PIN structure adopts an ordered conformation closely related to that of ex-PIN Monomer-B and substantially different from that of Monomer-A (Fig. 6D). This observation suggests the wing can undergo conformational exchange, perhaps to accommodate entry and exit of RNA substrates, adapt to differences in substrate sequence composition and secondary structure or to associate with ZAP.

The Nt-Arm contains 20 residues (K417–D436) that wrap around the core PIN domain (Fig. 6E). PISA analysis reveals an extensive interface that buries 890 Å^2^ of surface containing salt bridge, hydrogen bonds and hydrophobic interactions. The large extent of interaction suggests that it forms an integral part of the fold and is likely required to maintain the domain stability. Examination of the interface reveals the Nt-Arm largely interacts with residues in the PIN domain α2–α5 helical wing packing into a groove between α3 and α5. Within the interface, the Nt-Arm backbone amide and carbonyls make salt bridge and hydrogen bond interactions with the R460, Q467, W470, R475, and K500 side chains extending from α3 and α5 (Fig. 6F). In addition, the packing of Nt-Arm apolar side chains L420, F424, L426, and L428 with hydrophobic side chains A463, I464, V466, and F497, L501, L506 that otherwise would be exposed on α3 and α5 further contribute to the interface. These extensive Nt-Arm–PIN interactions lend support to the notion that, although the Nt-Arm is absent from the PIN domains of the ZC3H12 family of ribonucleases, for KHNYN and likely N4BP1 the Nt-Arm is required intrinsically for domain stability and catalytic activity and potentially for regulation of function, for example, through interaction with other protein partners.

Further structural alignment searches of ex-PIN against the PDB using the DALI server revealed the PIN domains of ZC3H12 proteins as top matches (*Z*-scores > 22) with the RNase-P nuclease domains from PRORPs [41] and mammalian mitochondrial tRNA processing enzymes identified with lower similarity scores (*Z* > 9 < 12) (Supplementary Table S4).

**Figure 7.**
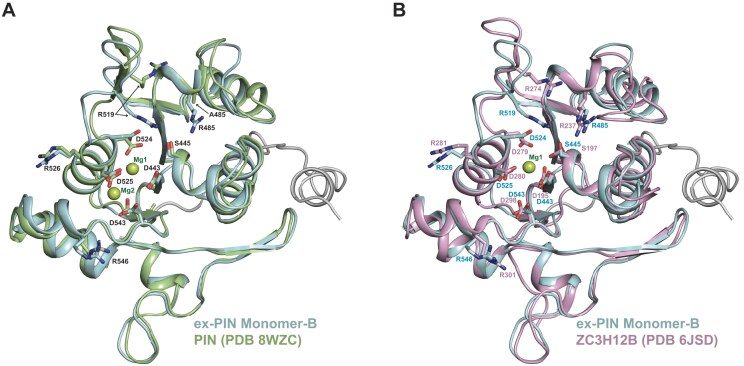
KHNYN PIN domain active site. DALI 3D structural alignment of the ex-PIN active site with (**A**) PIN (PDB: 8WZC). RMSD 1.2 Å over 161 Cα atoms and (**B**) the ZC3H12B PIN domain (PDB: 6SJD) RMSD (1.6 Å over 161 Cα atoms). Protein backbones, in cartoon, are colored cyan (ex-PIN; Monomer-B), green (PIN), and pink (ZC3H12B). The ex-PIN Nt-Arm is shown in gray. Conserved residues surrounding the active sites including tetra-Asp motifs (KHNYN; D443, D524, D525, and D543) and (ZC3H12B; D195, D279, D280, and D298) are shown in sticks. Mg ions bound in the PIN and ZC3H12B structures are shown as pale lemon-green spheres.

The DALI analysis also facilitated a comparison of Mg^2+^ ion occupancy in PIN active sites containing; none, KHNYN ex-PIN; one, ZC3H12B (PDB: 6SJD) or two, KHNYN PIN (8WZC), Mg^2+^ ions. A similarly positioned Mg^2+^ ion (Mg1) is present in both the KHNYN PIN and ZC3H12B PIN structures co-ordinated by D524 (D279) and D525 (D280) [36, 43], whilst a second Mg^2+^ (Mg2) is observed only in the RNA-bound KHNYN PIN complex co-ordinated by D525 and D543 [36]. Pairwise structural alignment of these three active sites reveals a conserved central pocket surrounded by β1, β2, α2, and α6 containing the tetra-Asp motif and places the Asp quartets, as well as conserved arginine and serine residues in near perfect alignment (Fig. 7A and B). In addition, apart from a small rotation of the D524 side chain in the apo-structure toward the Mg1 metal ion in the complexes it appears that co-ordination of either one or two Mg^2+^ ions does not result in large active-site conformational rearrangements. These observations suggest the active site is preformed to accept metal ions and an RNA substrate rather than metal- and RNA-binding requiring an induced fit. However, given only Mn^2+^ stimulates catalysis it is possible that there may be further movements in metal-binding and catalytic residues only when Mn^2+^ is bound. Notably, although largely absent in the apo ex-PIN structure, both complex structures contain a large number of water-mediated interactions that bridge between active site side chains and the bound Mg^2+^ and RNA [36, 43]. These water networks may be important for the PIN to accommodate different RNA sequence and backbone conformations in Mg^2+^ complexes. Alternatively, as we observe no substantial catalytic activity with Mg^2+^, it is possible that bound Mn^2+^ may produce a more catalytically active conformation where water-mediated interactions are replaced by direct metal-ion and side chain interactions with the RNA.

### The ex-PIN N-terminal arm is required for KHNYN restriction activity

**Figure 8.**
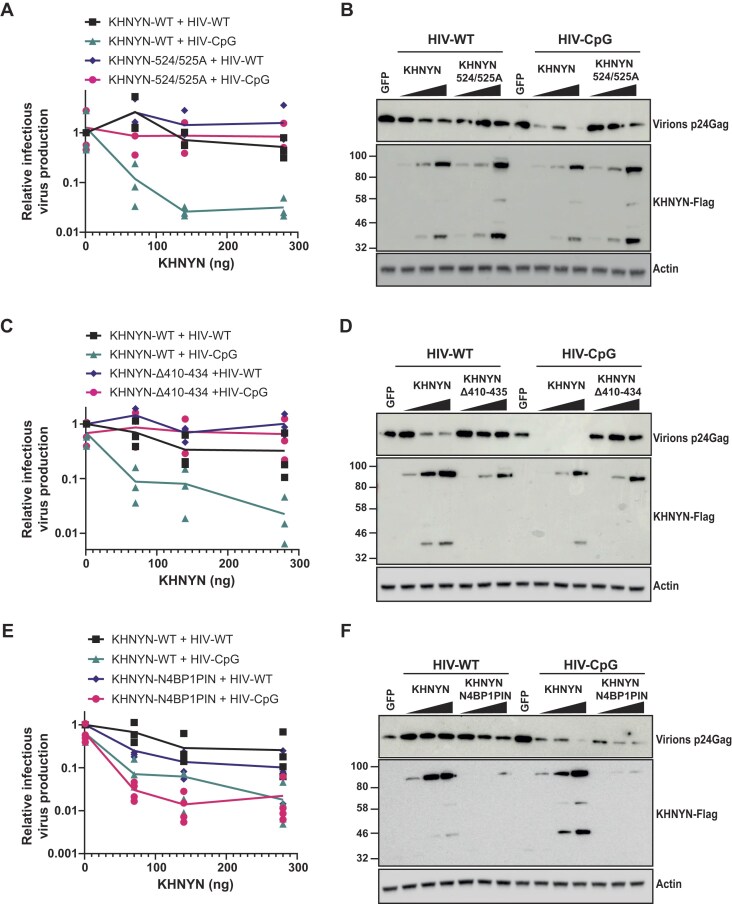
Ex-PIN domain mutants and antiviral activity. Infectious virus production from KHNYN CRISPR KO HeLa cells co-transfected with pHIV-WT or pHIV-CpG and increasing amount of CRISPR-resistant (**A**) WT pKHNYN or pKHNYN(524/525A) (**C**) WT pKHNYN or pKHNYN(Δ410–434) and (**E**) WT pKHNYN or pKHNYN(N4BP1PIN). Each data point represents the value from an independent experiment normalized to the value obtained for pHIV-WT in the absence of KHNYN. Three of the four replicates for KHNYN-WT + HIV-WT and KHNYN-WT + HIV-CpG are the same datapoints in panels (C) and (E). (**B, D**, and **F**) Representative western blots of the protein levels in restriction assays, (B) WT KHNYN and KHNYN(524/525A), (D) WT KHNYN and KHNYN(Δ410–434) and (F) WT KHNYN and (KHNYN-N4BP1PIN). Membranes are probed with antibodies to detect HIV-1 p24^Gag^ in virions, KHNYN-FLAG expression in the transfected cell lysate and Actin as the loading control. Membranes were also probed to detect the of level of HIV-1 Gag and Env in producer cell lysates, presented in Supplementary Fig. S2.

To determine the role of the ex-PIN Nt-Arm in antiviral activity, we used HIV-1 as a model system to study ZAP-KHNYN-mediated restriction [1, 12, 18, 70–72]. Wild-type HIV-1 (HIV-WT) is highly depleted in CpG dinucleotides and is therefore not efficiently targeted by ZAP. However, when a specific region in HIV-1 *env* is engineered to contain additional CpGs through synonymous mutations (HIV-CpG), the virus becomes ZAP-sensitive [12, 73–76]. This allows matched ZAP-resistant and ZAP-sensitive viruses to be tested to determine how ZAP and its cofactors restrict viral replication and evaluate antiviral activity specificity. To test the functional role of the ex-PIN Nt-Arm, an expression plasmid encoding CRISPR resistant KHNYN with the 25 residue ex-PIN extension deleted (pKHNYN(Δ410–434)) was co-transfected into KHNYN CRISPR knockout HeLa cells with plasmids encoding HIV-WT or HIV-CpG. As a control, a tetra-Asp motif catalytically dead mutant, pKHNYN (524A/525A), was also tested. Both proteins were expressed but lost substantial antiviral activity against HIV-CpG (Fig. 8A-D and Supplementary Fig. S2), indicating that the N-terminal extension to the PIN domain is required for antiviral activity.

N4BP1 and KHNYN contain three conserved domains, the ex-diKH domain, the PIN domain and the CUE^like^ domain. To determine whether these domains are functionally equivalent, we have previously made chimeric KHNYN proteins in which the ex-diKH domain or the CUE^like^ domains were swapped with the homologous domain in N4BP1 and measured their antiviral activities [33]. This showed that the ex-diKH domains from N4BP1 and KHNYN are functionally equivalent while the KHNYN CUE^like^ domain cannot be replaced by that of N4BP1, potentially because of the KHNYN nuclear export signal lacking in N4BP1 or ZAP binding residues that differ between the two proteins [19, 33]. To determine whether the PIN domains from N4BP1 and KHNYN are functionally equivalent, we replaced the KHNYN ex-PIN domain with that of N4BP1 (pKHNYN N4BP1PIN). This chimeric protein had high antiviral activity on both HIV-WT and HIV-CpG (Fig. 8E), even though it was expressed at low levels (Fig. 8F and Supplementary Fig. S2). These data show that the nuclease activity of the N4BP1 ex-PIN can efficiently complement KHNYN restriction. One possibility for the activity of this chimeric protein against both viruses is that it lacks the repression to ensure that it only cleaves RNA when it has been recruited by ZAP molecules clustered on a target transcript.

## Discussion

### The ex-PIN N-terminal arm

By contrast to recent publications [35, 36, 38] that showed the core PIN domain had only limited endonuclease activity with no sequence specificity at high enzyme to substrate ratios, we find the ex-PIN is highly active in the presence of Mn^2+^. Our structural studies have revealed the KHNYN ex-PIN Nt-Arm, an N-terminal region that traverses the KHNYN PIN domain and found only in the closely related PIN domain of N4BP1 and not the other more distantly related PINs of ZC3H12 and PRORP proteins. The requirement for this elaboration may simply be for domain stability as we found inclusion of the Nt-Arm greatly increased KHNYN PIN protein solubility in our *E. coli* expression system. Moreover, the Nt-Arm makes numerous favorable interactions with PIN side chains that would otherwise be exposed on the domain surface. In addition, our antiviral activity data support a Nt-Arm functional role as deletion of the Nt-Arm results in a loss of KHNYN–ZAP restriction. These observations suggest that the N4BP1 and KHNYN ex-PIN domains that have diversified in structure and sequence from other PIN domains and contain the Nt-Arm as requirement for their biological function. One possibility is that in ex-Pin the Nt-Arm may have replaced a protein binding partner required for function of other PIN domain family members but no longer required by KHNYN and N4BP1. However, given the proximity to the endonuclease active site and the requirement for ZAP to directly recruit KHNYN, although the KHNYN CUE^like^ domain is sufficient to bind to the ZAP RNA-binding domain [19], it is possible the Nt-Arm is an adaptation that is required to co-operate with ZAP or other cellular factors to regulate the mechanism of restriction but this has yet to be tested.

### Sequence specificity of KHNYN endonuclease

Our hydrolysis data employing RNA and DNA substrates has shown that whilst the KHNYN ex-PIN has no activity against DNA substrates, it hydrolyses a variety of single-stranded RNAs with distinct banding patterns indicative of endonuclease activity rather than exonuclease activity. The contribution of the CUE^like^ domain appears negligible suggesting that, although the CUE^like^ domain is responsible for coupling of nuclease activity to ZAP CpG RNA binding [19, 38], it does not directly influence the intrinsic ex-PIN endonuclease activity. Interestingly, a chimeric protein in which the N4BP1 ex-PIN replaced the KHNYN ex-PIN domain had high antiviral activity. It is not known how KHNYN is regulated to specifically cleave ZAP-targeted RNA but given the high activity on both HIV-WT and HIV-CpG for the chimeric protein, post-translational modifications in the PIN domain or interaction with other partners, which may differ between KHNYN and N4BP1, are two possibilities. Notably, this regulation is observed in ZC3H12A where activity is controlled by both post-translational modifications and protein-protein interactions [77].

In terms of sequence specificity, our data show that the ex-PIN domain shows a selectivity for RNAs containing ApC, ApA, and UpA dinucleotides and the capacity to cleave 33mer RNAs within three nucleotides of the 3′-end and four nucleotides of the 5′-end. Of the RNAs tested, seventeen cleavage sites were identified in total with fifteen accounted for by hydrolysis at seven ApC sites, three ApA sites, and five UpA sites with the only other cleavages at one ApU and one UpC site. Some susceptible RNAs also contained runs of U-bases that interspersed ApC, ApA, and UpA dinucleotides that were refractory to cleavage. Notably, the CpG-rich RNA containing six CpG dinucleotides was also highly resistant to KHNYN ex-PIN endonuclease activity revealing that, although CpG-rich RNA is largely targeted through ZAP recruitment, the cleavage sites likely lie in surrounding sequences that include many ApC, ApA, and UpA dinucleotides. Previous attempts to identify and rationally design a ZAP Responsive Element (ZRE) have not defined all the requirements but do show that an array of 15 or more CpG dinucleotides spaced at 12–30 nucleotide intervals in the context of surrounding A-U rich sequence constitute a ZRE that is sufficient for ZAP-dependent restriction of HIV-1 [78]. Given we now observe specificity in KHNYN endonuclease activity, it is also possible that the proximity to susceptible surrounding ApC, ApA, and UpA sites might also be a requirement for a functional ZRE. Of these dinucleotides UpA is broadly supressed in RNA virus genomes [79]. Additionally, the A-rich dinucleotide bias of KHNYN cleavage sites is consistent with the targeting of the ZAP-KHNYN to regions of ssRNA bound by ZAP [70, 80] because these ssRNA regions in many types of viruses are enriched in adenosine [81]. Thus, the A-rich bias in the cleavage specificity of the KHNYN ex-PIN domain may be an evolutionary adaptation to optimally cleave within with single-stranded regions of target RNAs.

### Manganese stimulated catalysis and innate immunity

Comparison of our KHNYN apo ex-PIN and Mg-bound PIN structures revealed there are no large conformational effects at the active site tetra-Asp motif upon Mg^2+^ co-ordination. Additionally, in the KHNYN PIN-Mg^2+^-RNA structure many of the side-chain interactions that coordinate the active site metal ions and RNA are water bridged suggesting there is a prefixed catalytic conformation, incorporating a water network, regardless of whether the metals required for hydrolysis are bound. Alternatively, given our data demonstrate Mn^2+^ is strictly required for KHNYN endoribonuclease activity and that Mg^2+^ has an inhibitory effect it is possible that introduction of Mn^2+^ might induce further conformational effects at the active site to produce a configuration more stimulatory to catalytic hydrolysis.

A variety of enzymatic hydrolytic reactions are stimulated by both Mg^2+^ and Mn^2+^ [82–85] likely as a result of their similar ionic radii, p*K*a of water dissociation and ligand binding geometries [86, 87]. By contrast, our observations demonstrate that KHNYN ex-PIN is highly discriminatory with only Mn^2+^ able to support efficient endonuclease activity. Other studies have reported Mn^2+^ as a selective regulatory ion in innate antiviral immunity required to restrict viral replication through multiple mechanisms. It has been shown that Mn^2+^ is released into the cytosol from the Golgi apparatus and mitochondria after viral infection [88]. This activates the cGAS–STING pathway through Mn^2+^ stimulation of cGAS activity [89] and enhancing cGAMP binding to STING [88, 90] which promotes the antiviral response to DNA viruses. In addition, Mn^2+^ regulates the antiviral response against both RNA and DNA viruses by promoting phosphorylation of TBK1 and downstream transcription factors, which increases type I and type III interferon induction and ISG expression [90–92]. These observations combined with our data support the hypothesis that Mn^2+^ could regulate antiviral innate immunity at the level of pattern recognition receptor signalling and direct restriction of viral replication by ISGs such as ZAP. Furthermore, the stringent requirement for Mn^2+^ in the activation of KHNYN endoribonuclease activity suggests Mn^2+^ stimulation of KHNYN could also be employed as a regulatory mechanism. In this way, ZAP directed KHNYN endoribonuclease activity would be licensed through the release of Mn^2+^ only after viral infection preventing off-target RNA decay.

## Supplementary Material

gkaf1360_Supplemental_File

## Data Availability

The co-ordinates and structure factors for the KHNYN ex-PIN X-ray structure have been deposited at the Protein Data Bank (PDB) (https://www.rcsb.org/) with the accession code ID: 9S2D. All other data are contained within the manuscript.

## Notes

Present address, University of Durham, Department of Biosciences, Durham DH1 3LE, UK
