# Supplementary material for: KHNYN is a manganese-dependent endoribonuclease required for ZAP-mediated antiviral restriction
Source: Nucleic Acids Res. 2025 Dec 17;53(22):gkaf1360. doi: 10.1093/nar/gkaf1360 (PMC12709174; doi:10.1093/nar/gkaf1360)
Supplement: gkaf1360_Supplemental_File [file gkaf1360_supplemental_file.pdf]

KHNYN is a manganese-dependent endoribonuclease required for ZAP mediated antiviral restriction.

Supplementary information

Supplementary Table 1. Primers for PCR-cloning and mutagenesis

**Construct**		**Primers (5' - 3')*[#]**
KHNYN PIN (P435-L591)	FWD	CAGGGACCCGGGCCGGATCTGCGTCATATTGTTATT
	REV	GCCAAGCTTTCACTCGAGTGGCAGAAATTCATCCAGTGTCGGACC
KHNYN ex-PIN (V410-P594)	FWD	CAGGGACCCGGTGTTACCGGTACGCAGCGTTTT
	REV	GGCACCAGAGCGTTACGGTTTTTTCAGAAATTCATCCAG
KHNYN ex-PIN-CUE$_{like}$ (V410-F678)	FWD	CAGGGACCCGGTGTTACCGGTACGCAGCGTTTT
	REV	GGCACCAGAGCGTTAAAAATTCAGTGACAGCAGCGCTTC
KHNYN (ex-PIN) D424A/D525A	FWD	CGTATTAGCAGCTAT**GCTGCA**CGCTTTATGGTTAAACTG
	REV	CAGTTTAACCATAAAGCG**TGCAGC**ATAGCTGCTAATACG
KHNYN (N4BP1-PIN) (pN4BP1)	FWD	ACGAGGCGCCCGAGGGGGCAACTTGGTTACTGGGGTTCAAAGGTTTC
	REV	GAGCCTTAGAAGACCCCTGTGTCCTACAGACTTCCTTCTGAAGAAACTC
KHNYN (N4BP1-PIN) (pMF52)	FWD	AGGACACAGGGGTCTTCTAAGG
	REV	CAAGTTGCCCCCTCGGGCGC
KHNYN (Δ410-434)	FWD	CCAGACCTCCGCCATATTGTCATTG
	REV	CAAGTTGCCCCCTCGGGC

*LIC or restriction sites used for cloning are underlined; [#]Mutagenized codons are highlighted and underlined

Supplementary Table 2. Protein purification buffers

Buffer	Composition
PIN Lysis	100 mM BTP pH 8.0, 150 mM NaCl, 0.5 mM TCEP, 5% (w/v) glycerol, 10 mM $MgCl_2$, 10 mM imidazole, 0.2% (v/v) Triton X-100, 1x cOmplete EDTA-free protease inhibitor tablet per 50 mL and 2.5 units/mL Benzonase Nuclease
PIN Wash (75 mL)	100 mM BTP pH 8.0, 500 mM NaCl, 0.5 mM TCEP, 10 mM $MgCl_2$, 25 mM imidazole, 5 mM ATP
PIN Elution	100 mM BTP pH 8.0, 250 mM NaCl, 0.5 mM TCEP, 10 mM $MgCl_2$, 500 mM imidazole
PIN GF	50 mM BTP pH 8.0, 200 mM NaCl, 0.5 mM TCEP, 5 mM $MgCl_2$
ex-PIN Lysis	100 mM BTP pH 8.0, 300 mM NaCl, 0.5 mM TCEP, 5% (w/v) glycerol, 10 mM $MgCl_2$, 1x cOmplete EDTA-free protease inhibitor tablet per 50 mL and 20 units/mL Salt Active Nuclease
ex-PIN Wash 1 (150 mL)	100 mM BTP pH 8.0, 750 mM NaCl, 0.5 mM TCEP, 5% (w/v) glycerol, 10 mM $MgCl_2$, 5 mM ATP
ex-PIN Wash 2 (75 mL)	100 mM BTP pH 8.0, 300 mM NaCl, 0.5 mM TCEP, 5% (w/v) glycerol, 10 mM $MgCl_2$
ex-PIN GF	50 mM BTP pH 8.0, 200 mM NaCl, 0.5 mM TCEP, 5% (w/v) glycerol, 2 mM $MgCl_2$
ex-PIN-CUE^{like} Lysis	100 mM BTP pH 6.0, 300 mM NaCl, 0.5 mM TCEP, 5% (w/v) glycerol, 10 mM $MgCl_2$, 1x cOmplete EDTA-free protease inhibitor tablet per 50 mL and 20 units/mL Salt Active Nuclease
ex-PIN-CUE^{like} Wash 1 (150 mL)	100 mM BTP pH 6.0, 750 mM NaCl, 0.5 mM TCEP, 5% (w/v) glycerol, 10 mM $MgCl_2$, 5 mM ATP
ex-PIN-CUE^{like} Wash 2 (75 mL)	100 mM BTP pH 6.0, 300 mM NaCl, 0.5 mM TCEP, 5% (w/v) glycerol, 10 mM $MgCl_2$
ex-PIN-CUE^{like} GF	50 mM BTP pH 6.0, 200 mM NaCl, 0.5 mM TCEP, 2 mM $MgCl_2$

Supplementary Table 3. Oligonucleotide RNA and DNA substrates

Name	Sequence*
RNA Env(48-58)CpG-rich	FAM-5′-GCAACGACGACGCUAUUUUGCGCAUCAGACGCG-3′
RNA Env(48-58) CpG-depleted	FAM-5′-GCAACCACCACUCUAUUUUGUGCAUCAGAUGCU-3′
RNA U-rich	FAM-5′-GUUUCUUUUACUCUUUGUUACUUUUCAGUUAAU-3′
RNA A-rich	FAM-5′-AUAACAAAUACACUAAAAUACAAAUCAGAAAAU-3′
RNA AU-rich	FAM-5′-GUAACUAAUACUCUAUUUUGUUAAUCAGAUAAU-3′
RNA UA-rich	FAM-5'-GUUUCUUUUAGUCUUUGUUAGUUUUCAGUUAAU-3′
RNA C-rich	FAM-5′-CUCCUCCCUACACUCCCCUCUCCCGCCGCCCCU-3′
dsDNA-70mer	FAM-5′-GTAAGTGCCGCGGTGCGGGTGCCAGGGCGTGCCCTTGGGCTCCCCGGGCGCGTACTCCACCTCATGCATC-3′
	3′-CATTCACGGCGCCACGCCCACGGTCCCGCACGGGAACCCGAGGGGCCCGCGCATGAGGTGGAGTACGTAG-5′
dsDNA 5'-25mer	FAM-5′-GTAAGTGCCGCGGTGCGGGTGCCAG-3′
	3′-CATTCACGGCGCCACGCCCACGGTCCCGCACGGGAACCCGAGGGGCCCGC-5′
dsDNA 3'-70mer	FAM-5′-GTAAGTGCCGCGGTGCGGGTGCCAGGGCGTGCCCTTGGGCTCCCCGGGCGCGTACTCCACCTCATGCATC-3′
	3′-CATTCACGGCGCCACGCCCACGGTCCCGCACGGGAACCCGAGGGGCCCGC-5′
ssDNA 70mer	FAM-5′-GTAAGTGCCGCGGTGCGGGTGCCAGGGCGTGCCCTTGGGCTCCCCGGGCGCGTACTCCACCTCATGCATC-3′
ssDNA 25mer	FAM-5′-GTAAGTGCCGCGGTGCGGGTGCCAG-3′

RNA CpG dinucleotides, red – underlined; phosphorothioate protected nucleotides, blue.

Supplementary Table 4. DALI structural similarity search

Match	PDB code	Z score	Rmsd (Å)	Cα aligned	Total residues	Seq ID (%)
ZC3H12C (MCPIP3)	7NDH	24.6	1.5	162	169	56
ZC3H12A (MCPIP1)	5H9W	24.0	1.4	157	162	55
ZC3H12B (MCPIP2)	6SJD	23.0	1.6	161	168	55
PRORP1	4G23	11.4	2.9	133	476	21
PRORP2	5DIZ	11.0	2.9	129	474	19
RnaseP	8CBK	9.7	2.8	132	470	14
RnaseP	4XGL	9.0	2.7	111	342	14

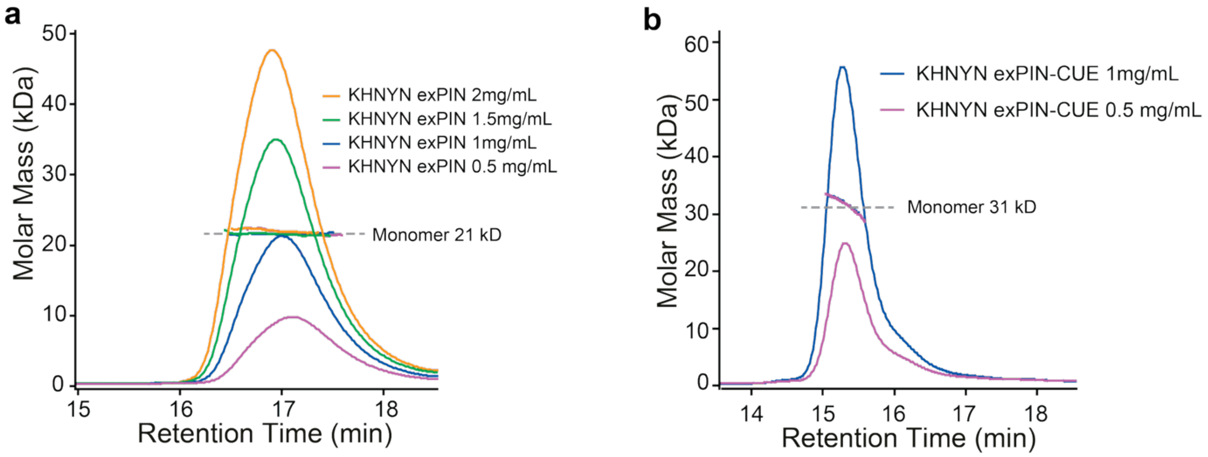

Supplementary Figure 1. Solution molar mass of KHNYN ex-PIN, ex-PIN-CUE^{like} and ZAP RBD. SEC-MALLS analysis of (**a**) KHNYN ex-PIN and (**b**) KHNYN ex-PIN-CUE^{like}. In each panel, the differential refractive index (dRI) is plotted against column retention time for the indicated sample loadings. The molar mass, determined at 1-second intervals throughout the elution of peaks, is plotted as points. The monomer molar masses of KHNYN ex-PIN and ex-PIN-CUE^{like} are indicated by the grey dashed lines.

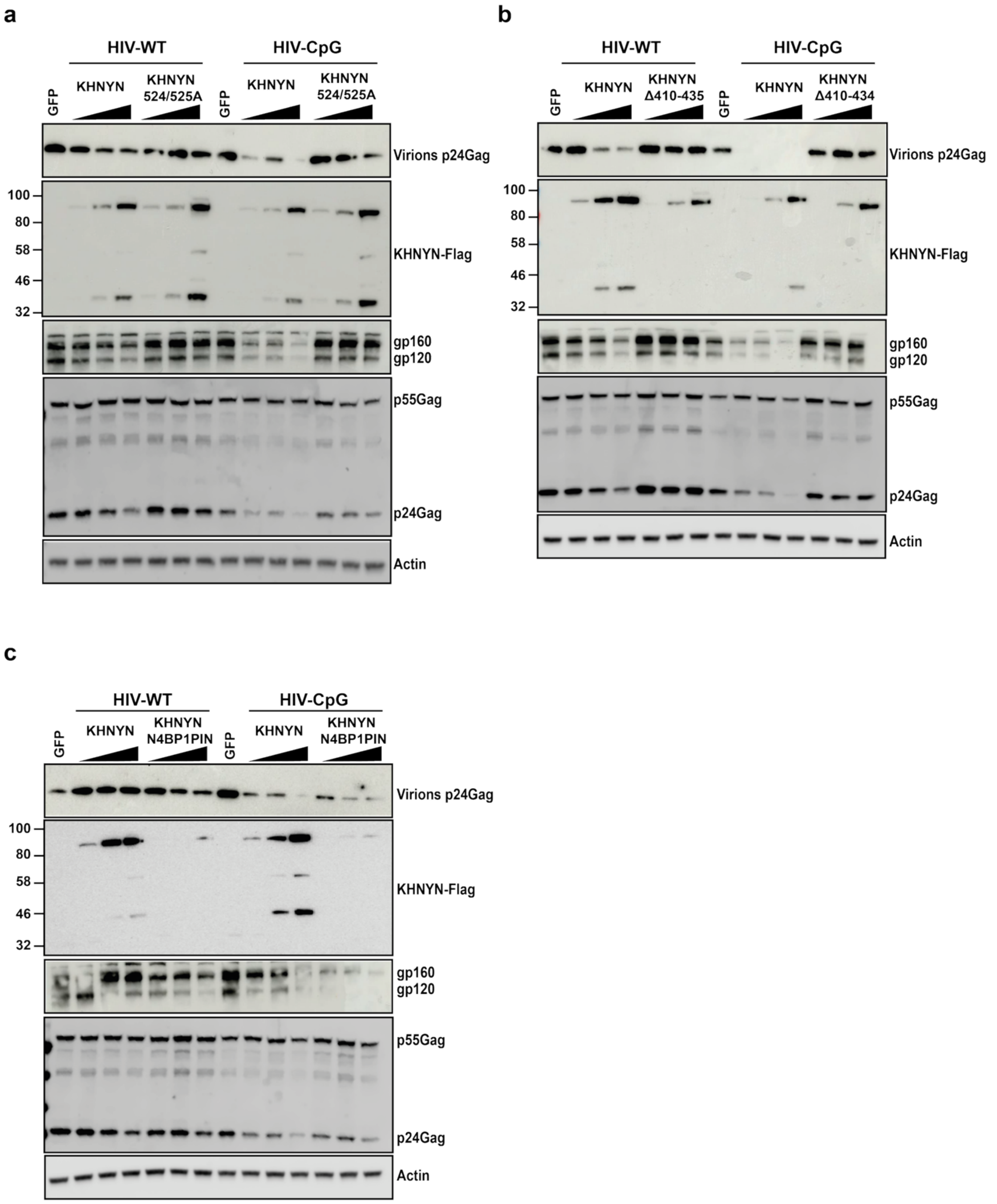

Supplementary Figure 2. Immunoblot analysis of KHNYN expression. Representative Western blots of the protein levels in restriction assays presented in **Figure 8**. (**a**) WT KHNYN and KHNYN(524/525A), (**b**) WT KHNYN and KHNYN(Δ410-434) and (**c**) WT KHNYN and (KHNYN-N4BP1PIN). Membranes are probed with antibodies to detect the of level KHNYN constructs and HIV-1 Gag and Env in producer cell lysates, HIV-1 p24 in virions and an actin loading control.
